# Evolution of non-kin cooperation: social assortment by cooperative phenotype in guppies

**DOI:** 10.1098/rsos.181493

**Published:** 2018-12-26

**Authors:** Josefine Bohr Brask, Darren P. Croft, Mathew Edenbrow, Richard James, Bronwyn H. Bleakley, Indar W. Ramnarine, Robert J. P. Heathcote, Charles R. Tyler, Patrick B. Hamilton, Torben Dabelsteen, Safi K. Darden

**Affiliations:** 1 Centre for Research in Animal Behaviour, College of Life and Environmental Sciences, University of Exeter, Exeter EX4 4QG, UK; 2 Centre for Networks and Collective Behaviour and Department of Physics, University of Bath, Bath BA2 7AY, UK; 3 Department of Biology, Stonehill College, Easton, MA 02357, USA; 4 Department of Life Sciences, The University of the West Indies, St Augustine, Trinidad and Tobago; 5 Department of Biosciences, College of Life and Environmental Sciences, University of Exeter, Exeter EX4 4QD, UK; 6 Behavioural Ecology Group, Department of Biology, University of Copenhagen, 2100 Copenhagen Ø, Denmark

**Keywords:** cooperation, assortment, guppy, social structure, social networks, predator inspection

## Abstract

Cooperation among non-kin constitutes a conundrum for evolutionary biology. Theory suggests that non-kin cooperation can evolve if individuals differ consistently in their cooperative phenotypes and assort socially by these, such that cooperative individuals interact predominantly with one another. However, our knowledge of the role of cooperative phenotypes in the social structuring of real-world animal populations is minimal. In this study, we investigated cooperative phenotypes and their link to social structure in wild Trinidadian guppies (*Poecilia reticulata*). We first investigated whether wild guppies are repeatable in their individual levels of cooperativeness (i.e. have cooperative phenotypes) and found evidence for this in seven out of eight populations, a result which was mostly driven by females. We then examined the social network structure of one of these populations where the expected fitness impact of cooperative contexts is relatively high, and found assortment by cooperativeness, but not by genetic relatedness. By contrast, and in accordance with our expectations, we did not find assortment by cooperativeness in a population where the expected fitness impact of cooperative contexts is lower. Our results provide empirical support for current theory and suggest that assortment by cooperativeness is important for the evolution and persistence of non-kin cooperation in real-world populations.

## 1. Introduction

Cooperation has for many years constituted a puzzle for evolutionary theory. Cooperative behaviours, defined as acts that benefit a receiver with a net cost to the actor, are found in many taxa but seemingly contradict the central prediction in classic evolutionary theory that behaviours must be fitness-increasing for actors in order to be selected for and maintained. While cooperation among kin can be explained by kin selection [1], the evolution and maintenance of non-kin cooperation is still not well understood. It has received extensive theoretical attention and various potential explanations have been proposed (e.g. [2–5]), but it is currently unknown how non-kin cooperation is actually maintained in nature.

A central insight from theoretical work is that assortment of cooperative acts is a fundamental prerequisite for the maintenance of cooperation (e.g. [6–10]). Assortment of cooperative acts entails an increased likelihood of cooperative acts to be met with cooperation and defective acts to be met with defection. Such assortment can generally be achieved in two ways. One is that individuals can use flexible conditional rules for when to cooperate that create assortment of cooperative acts among individuals (e.g. [3]). Another way is assortment of individuals in social space, where cooperative behaviour is a repeatable trait of the individual, and cooperative individuals associate and interact with each other disproportionately more than with defectors (e.g. [6]). Empirical research examining non-kin cooperation has focused on the first of these possibilities. However, there is growing evidence that individuals from a range of species show stability in their level of cooperativeness [11]. Furthermore, numerous studies have demonstrated that animal social network structures are non-random, with significant within-population heterogeneity in social tie strengths documented across species [12]. These findings suggest that assortment of individuals in social space based on their cooperativeness could potentially be promoting cooperation among non-kin in many species. Direct empirical investigations of social assortment by cooperativeness in natural populations of animals are therefore essential to understanding non-kin cooperation, but knowledge regarding the link between social structure and individual cooperativeness in animals is currently minimal.

In this study, we empirically investigated individual cooperativeness and its association with social structure in wild Trinidadian guppies (*Poecilia reticulata*). This freshwater fish is a classic model species in behavioural ecology and evolution [13] and specifically in the study of cooperation (e.g. [14–17]). In guppies, cooperative behaviour is expressed during predator inspection, a behaviour where individuals approach a predator and gain information regarding the threat it poses [18]. The inherent risk of this behaviour can be counteracted by individuals performing it cooperatively [16], but individuals that do not join inspections also gain information from inspectors, meaning that there is a temptation to defect. Because cooperative situations are connected to predation in this species, the expected fitness impact of cooperative situations differs between populations living under different predation regimes [13]. Guppies inspect more often with preferred shoaling partners, and cooperation seems to not be based on kinship in this species [19,20]. While guppies can adjust their level of cooperativeness to the cooperativeness level of predator inspection partners to some degree [15,17], previous work (with laboratory-reared male guppies descended from wild fish) suggests that they do have consistent cooperative phenotypes [15]. Here, we investigate the existence of cooperative phenotypes in wild guppies and how these phenotypes are linked to social network structure.

Our study has two parts. First, we investigate stability in individual cooperativeness (i.e. cooperative phenotypes) in eight populations by testing the within-individual repeatability of cooperativeness scores measured during standardized predator inspection assays. Second, we investigate links between individual cooperativeness and social structure. To this end, we quantify the social network of a population where the expected fitness impact of cooperative contexts (predator inspection events) is relatively high due to high-predation pressure from piscivorous fish. Furthermore, natural guppy populations experience considerably different predation pressures within short geographical distances [13], and this gives us a rare opportunity to assess these results in relation to those of a geographically close population where the expected fitness impact of predator inspection is relatively low due to low predation. For both populations, we also quantify genetic relatedness among individuals, to assess whether kinship could play a role for cooperation in these populations. Because of the larger expected impact of cooperative contexts on fitness in the high-predation population, we predicted that links between cooperativeness and social structure, and in particular assortment by cooperativeness, would be primarily expressed in this population.

## 2. Material and methods

### 2.1. Study populations and general methods

**Figure 1.**
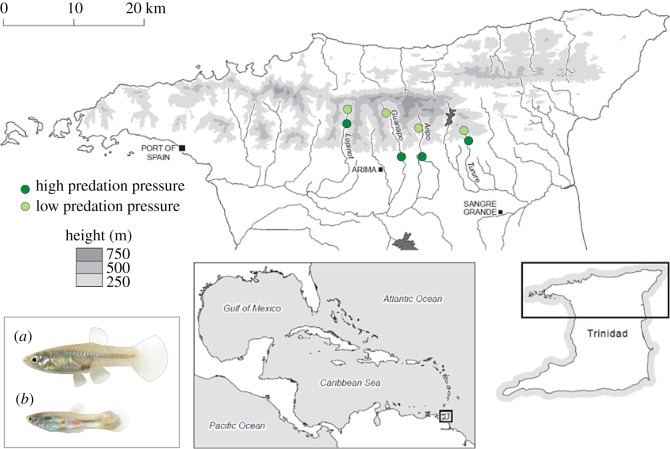
Map of the northern part of Trinidad with the positions of the eight guppy populations used for data collection indicated in green. A female (*a*) and male (*b*) Trinidadian guppy is shown.

Data were collected from February to August 2012 from eight wild populations of Trinidadian guppies living in four rivers in the Northern Mountain Range of Trinidad (the rivers Aripo, Turure, Guanapo and Lopinot; figure 1). In each river, one population living under high predation pressure (in the following named the HP population) and one living under low predation pressure (the LP population) were sampled. Low-predation sites were characterized by the absence of the major guppy predator *Crenicichla frenata*, which cannot cross larger waterfalls and therefore is not found in upstream pools [13].

Adult guppies were collected from river pools using seine nets and transferred the same day to the laboratory, where they were housed in large communal tanks (90 × 29 × 22 cm). In the laboratory, they were individually marked with Visible Implant Elastomer to allow visual identification ([21]; this marking does not influence shoaling behaviour [22]), their cooperativeness was assayed (described below), their body lengths were measured and tissue samples (non-destructive fin clips) were taken for relatedness analysis (details below). The fish were then returned to their pools of origin, and for two of the populations (those from the Aripo River), their social associations were sampled (described below). The fish collection and export of tissue samples were done under a permit from the Ministry of Agriculture, Land and Fisheries, Trinidad and Tobago.

### 2.2. Measuring cooperativeness

Cooperative behaviour was quantified by subjecting each individual to two predator inspection trials separated in time by 1 day. The trials took place in test tanks divided lengthwise into two identical test arenas by an opaque barrier (a schematic drawing of a test tank can be found in electronic supplementary material, appendix A). Two separate trials, one in each test arena, were carried out simultaneously. The two arenas were fully separated from each other, with no opportunity for information exchange between the fish in the two trials. In one end of each test arena was a refuge with a plastic plant, in the other end a predator enclosure, and between these an inspection lane. A transparent barrier separated the predator enclosure from the inspection lane. A mirror was placed on the inner side of the inspection lane, in parallel position to the lane. This was used to present a virtual inspection partner to the test fish, in the form of its own mirror image (as in e.g. [14,15]). This set-up standardizes the levels of cooperativeness of the partners relative to those of the test individuals and thereby removes confounding effects of variation in partner behaviour. To ensure that mirror trials are a valid method to measure cooperativeness, we investigated the relationship between cooperativeness measured in mirror trials and cooperativeness of the same focal individuals measured in trials with live partners (this analysis is described in electronic supplementary material, appendix B).

During mirror inspection trials, the test tank was filled with 10 cm of water. At the beginning of a trial, the test fish was placed in the middle of the inspection lane and allowed to settle for 5 min. During this time, the predator enclosure was hidden behind an opaque partition. The test fish was then guided into the refuge with a small dip net, the refuge was closed with a transparent partition and the opaque partition in front of the predator enclosure was lifted, making the predator visible to the test fish. After 1 min, the refuge partition was lifted, and a 5 min test period followed, during which the test fish could inspect the predator. All partitions were moved with a remote pulley system. To record the position of the test fish, we used the tracking software Ethovision XT v. 7 (Noldus Information Technology, Wageningen, The Netherlands) with a sampling rate of 30.1 frames s^−1^. For each trial, the average distance of the test fish from the refuge was calculated as a cooperativeness score (as in e.g. [15,23]), with fish moving farther away from the refuge and closer to the predator being the more cooperative, and an overall score of individual cooperativeness was obtained as the average of the two scores from the two trials.

### 2.3. Testing for cooperative phenotypes

To investigate whether guppies show stability in their individual levels of cooperativeness, i.e. cooperative phenotypes, we tested for significant repeatability in the two cooperativeness scores obtained from the mirror trials for each individual. We tested this for each of the eight populations, for all individuals and for the sexes separately, using one-way ANOVA tests, with cooperativeness scores as the dependent variable and individual identity as the independent variable. Owing to non-normality of data and transformation not giving satisfactory distribution, we used a permutation procedure to estimate the *p*-values. We applied the *aovp* method in the *lmPerm* R-package [24], with *Ca* set to 0.001, *maxIter* set to 10^9^ and *nCycle* set to 1 (see [24]). To account for multiple testing, we used a false discovery rate (FDR) control procedure to determine the significance of the tests [25]. We applied the control procedure to each of the three batches of tests on the eight populations (all individuals, females and males).

To quantify the repeatability in each population, repeatability scores were calculated by the formula [26]:

$$\mathrm{Repeatability}=\;\frac{(MS_{A}-MS_{W})/n_{0}}{MS_{W}+(MS_{A}-MS_{W})/n_{0}}$$

where MS*_A_* and MS*_W_* are the mean square among and within individuals given by the ANOVAs, and *n*_0_ is the number of measures taken for each individual; that is, in our case *n*_0_ = 2. The repeatability measure takes the maximum value of 1 if there is perfect repeatability, i.e. if there is no within-individual variation.

### 2.4. Measuring social structure

For two populations (Aripo HP and LP), social association data were collected from the marked fish in their natural pools over a period of 6 days, starting the day the fish were returned to the pools. Shoal compositions were sampled five times a day between 08.30 and 15.00 by photographing shoals from a marked transect along the riverside. All parts of the pool were visible from the transect. An individual was defined as belonging to a shoal if it was within four body lengths of any member of the shoal [27]. At least 25 min passed from the end of a sample to the beginning of the next, and because shoal composition changes on a time scale of seconds [27], association data from different samples were considered to be independent.

A weighted social association matrix was constructed for each of the two populations by applying a simple ratio association index (SRI; [28,29]). The edge weight for each dyad was calculated as the number of samples where the dyad was observed shoaling together, out of all samples where at least one of them was observed. This gave an edge weight between zero (the dyad was never observed in the same shoal) and one (the dyad was always observed in the same shoal). Only individuals observed more than three times were included in the association matrices to increase the precision of the association estimates.

### 2.5. Genotyping and relatedness analysis

We calculated the relatedness of individuals in the Aripo HP and LP populations to be able to investigate the role of kinship in social structure (as described in the next section). To calculate relatedness, we used 12 published microsatellite loci for each population (13 in total, see table in electronic supplementary material, appendix C for details). DNA extraction and amplification followed the procedure described in [20]. Briefly, following a Chelex extraction, 16 microsatellite loci were amplified in three polymerase chain reaction multiplexes, and genotypes were determined using the fragment analysis on CEQ 8000 (Beckman Coulter, Fullerton, USA). We calculated observed and expected heterozygosity using GENALEX v. 6.5 [30], and deviations from Hardy–Weinberg expectations and linkage between the microsatellite loci using GENEPOP v. 4.2 [31,32]. The Pr171 and PP_H2 loci deviated from Hardy–Weinberg expectations for both populations, as did G43 for the LP population and Pre8 for the HP population, and they were excluded from the relatedness analysis for the relevant populations. Relatedness was calculated using COANCESTRY [33].

### 2.6. Investigating the relationship between cooperative phenotype and social structure

To create null models of the two social networks, permuted versions of each of the association matrices were constructed with the following data stream permutation procedure. For each sample, a binary association matrix of the observed individuals was constructed in which each dyad got an association of one if they were observed in the same shoal within the sample and zero if they were not observed in the same shoal within the sample. The node labels of this matrix were then randomly permuted. From the permuted matrices of all the samples of the population, an SRI association matrix was then constructed as described above. This procedure randomizes association patterns while preserving the original shoal sizes, and for each individual the number of samples that it was observed in. Permuted networks mentioned below were all created with this procedure, unless stated otherwise.

To investigate the association between cooperative phenotype and social structure, we used the overall scores of individual cooperativeness from the mirror trials. We tested for assortment and disassortment by cooperativeness in the two populations. As assortment by cooperativeness could potentially arise as a by-product of assortment by body length and sex [22,27,34], we also tested for assortment (and disassortment) by these traits, as well as for correlations between cooperativeness and body length and differences in cooperativeness between sexes. Assortment and disassortment by cooperativeness, body length and sex, based on edge weights, were tested in each population by calculating the weighted assortativity coefficient *r^w^* [35] for the real network and comparing this to weighted assortativity coefficients calculated from 10 000 permuted versions of the network. Using two-tailed tests, we considered the test result to be significant if the real value was among the 2.5% highest or lowest of these values. Assortment and disassortment by cooperativeness based on the presence and absence of edges was calculated with a similar procedure, using the unweighted assortativity coefficient *r^u^*. For cooperativeness, we also tested for assortment and disassortment within the sexes for each population. All assortativity coefficients were calculated with the *assortnet* R-package [36]. As cooperativeness scores were not normally distributed, we tested for correlations between body length and cooperativeness in the two populations by non-parametric Spearman's rank correlation tests, and for differences between sexes in average cooperativeness by non-parametric Mann–Whitney U-tests.

It may be expected that any observed assortment by cooperativeness would be driven by individuals of high cooperativeness, as they should benefit more from having strong ties to each other than individuals of lower cooperativeness. To investigate whether strong social ties were primarily found among more cooperative individuals, for each of the two populations we tested for a correlation between the cooperativeness of all dyads (i.e. the sum of the cooperativeness of the two individuals) and their edge weight. We compared the Pearson correlation coefficient based on the real values with corresponding correlation coefficients calculated for 10 000 versions of the network where the edge weights had been randomly permuted. Using two-tailed tests, we considered the test result to be significant if the real value was among the 2.5% highest or lowest of the values from the networks with permuted edge weights.

The presence of social assortment by kinship in the populations would suggest that cooperation could be explained by kin selection [1]. We tested for assortment by kinship in each network by calculating the Pearson correlation coefficient between the edge weights of all dyads and their relatedness, and comparing this correlation coefficient to corresponding correlation coefficients calculated for 10 000 permuted versions of the network. Using two-tailed tests, we considered the test result to be significant if the real value was among the 2.5% highest or lowest of the values from the permuted networks.

In the following, values denoted *perm* are medians of null distributions of test values obtained from permuted data.

## 3. Results

### 3.1. Testing for cooperative phenotypes

Cooperativeness shown by individuals in trials with mirror-image partners was significantly correlated with their cooperativeness in live-partner trials (electronic supplementary material, appendix B), validating the use of mirror trials as a means to measure cooperativeness in guppies. Seven of the eight populations showed significant repeatability of cooperativeness scores within individuals when both sexes were included (table 1). This seemed to be mostly driven by females, with six populations showing significant repeatability for females, whereas for males significance was found in two populations (with four others showing a tendency towards significance; table 1). Both males and females of the Aripo HP population showed significant repeatability, whereas for Aripo LP only females showed significance, suggesting that the overall significant repeatability in Aripo LP was primarily due to females.

**Table 1.** Results of tests for repeatability of individual cooperativeness in eight populations of wild guppies living under high (HP) and low (LP) predation pressure. Significance was determined after controlling for multiple testing (FDR control, see Material and methods). Significant results are in bold.

individuals included	river	population	*n*	*F*	*p*-value	repeatability	significant after FDR control?
all	Aripo	HP	105	2.48	<0.0001	0.43	**yes**
		LP	144	1.73	0.0006	0.27	**yes**
	Turure	HP	97	2.47	<0.0001	0.42	**yes**
		LP	139	1.20	0.14	0.09	no
	Guanapo	HP	136	1.69	0.0012	0.26	**yes**
		LP	144	2.15	<0.0001	0.36	**yes**
	Lopinot	HP	136	1.44	0.017	0.18	**yes**
		LP	146	1.57	0.0036	0.22	**yes**
females	Aripo	HP	53	2.18	0.0035	0.37	**yes**
		LP	74	1.9	0.0042	0.31	**yes**
	Turure	HP	48	4.17	<0.0001	0.61	**yes**
		LP	72	1.34	0.11	0.15	no
	Guanapo	HP	73	1.92	0.0031	0.32	**yes**
		LP	73	2.24	0.0004	0.38	**yes**
	Lopinot	HP	74	1.48	0.048	0.19	no
		LP	74	1.57	0.028	0.22	**yes**
males	Aripo	HP	52	2.86	0.0002	0.48	**yes**
		LP	70	1.38	0.094	0.16	no
	Turure	HP	49	1.64	0.044	0.24	no
		LP	67	0.98	0.53	−0.01^a^	no
	Guanapo	HP	63	1.45	0.074	0.18	no
		LP	71	1.87	0.0051	0.30	**yes**
	Lopinot	HP	62	1.37	0.11	0.15	no
		LP	72	1.43	0.066	0.18	no

^a^Negative repeatability values occur when *F* < 1 [26].

### 3.2. Investigating the relationship between cooperative phenotype and social structure

**Figure 2.**
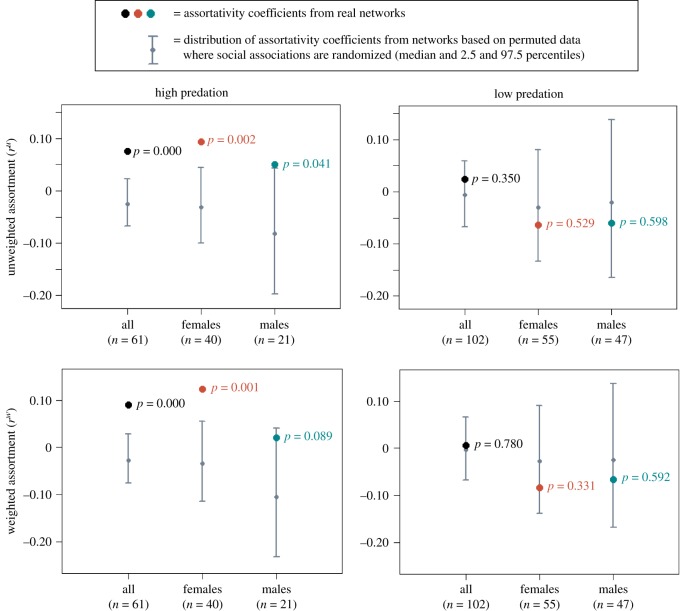
Results from tests of social assortment by cooperativeness in two wild guppy populations living under high and low predation, respectively. The results are for assortment based on the presence and absence of edges (unweighted assortment) and assortment based on edge weights (weighted assortment), for all individuals together and the sexes separately.

We found significant assortment by cooperativeness in the Aripo HP population both when assortment was based on edge weights (i.e. weighted; figure 2) and when it was based on the presence and absence of edges (i.e. unweighted; figure 2). Assortment results are given in tabular form in electronic supplementary material, appendix D. No significant assortment or disassortment by cooperativeness was found in the Aripo LP population, including within the sexes (figure 2). For the weighted assortment, the significant result for the HP population seemed to be driven mostly by females, who showed significant within-sex assortment, while males showed a tendency towards significance (figure 2). Both sexes showed significant assortment when only the presence and absence of edges were considered (figure 2). This suggests that both sexes in the HP population assort by cooperativeness, and within males the assortment is driven by presence rather than strength of associations (although it should be noted that the sample size of males is lower than that of females and could have contributed to the lack of significance in weighted assortativity).

While the HP population was assorted by body length (*n* = 61, $r^{w}=0.17$, $r_{\mathrm{perm}}^{w}=0.00$, *p* = 0.00), there was no significant relationship between body length and cooperativeness (*n* = 61, *r_s_* = −0.19, *p* = 0.15). Also, the HP population was not significantly assorted by sex (*n* = 61, $r^{w}=0.02$, $r_{\mathrm{perm}}^{w}=-0.03,$ *p* = 0.11) and did not show any significant difference in cooperativeness between sexes (*n* = 40 females 21 males, *W* = 413, *p* = 0.92). The observed assortment by cooperativeness in this population is therefore unlikely to have arisen as a by-product of assortment by body length or sex. The LP population was assorted by both body length (*n* = 102, $r^{w}=0.38,$ $r_{\mathrm{perm}}^{w}=0.03,$ *p* = 0.00) and sex (*n* = 102, $r^{w}=0.39$, $r_{\mathrm{perm}}^{w}=0.05,$ *p* = 0.00), and here we found a significant negative correlation between cooperativeness and body length (*n* = 102, *r_s_* = −0.21, *p* = 0.037) and a significant difference between the sexes in cooperativeness, with the males being more cooperative (*n* = 55 females 47 males, *W* = 1729, *p* = 0.0034).

We did not find any significant correlation between the summed cooperativeness of dyads and their edge weight in either population (HP: *n* = 1830 dyads, *r* = 0.00, *r*_perm_ = 0.00, *p* = 0.83; LP: *n* = 5151 dyads, *r* = 0.02, *r*_perm_ = 0.00, *p* = 0.16), meaning that there is no evidence for assortment by cooperativeness being driven by individuals of high cooperativeness.

No significant correlation between edge weights and relatedness was found in either population (HP: *n* = 61, *r* = −0.02, *r*_perm_ = −0.01, *p* = 0.50; LP: *n* = 102, *r* = 0.00, *r*_perm_ = 0.00, *p* = 0.94), indicating that social associations are not based on kinship.

## 4. Discussion

The evolution and persistence of cooperation in structured populations has been investigated extensively with individual-based computer models that use a game-theoretic framework (reviewed in [37,38]). This body of research demonstrates that certain social network topologies and dynamics can maintain cooperation, by giving rise to assortment of cooperative acts (e.g. [39–45]). However, very little is known about the link between cooperation and social structure in real-world populations. In this study, we investigated this link in wild guppies. We found repeatability in cooperativeness in seven out of eight wild guppy populations, providing strong evidence that cooperative behaviour is a phenotypic trait in guppies, particularly in females. When investigating the social structure of a population where guppies live under a high risk of predation and cooperative contexts (predator inspection events) therefore are of relatively high importance for fitness, we found social assortment by cooperativeness. The main necessary condition for the persistence of cooperative behaviour, as assessed by theoretical studies (assortment of cooperative acts; e.g. [6–10]), is thus fulfilled and the result implies that social assortment by cooperativeness is playing a role in the maintenance of cooperation in this population. Congruent with our expectation, we did not find assortment by cooperativeness in a population living under low predation pressure. As with previous work in another guppy population [20], we found that relatedness was not important for social associations in either population, implying that cooperation cannot be explained here by kin selection. (A detailed discussion of the lack of kin assortment in guppies can be found in [20].) Together, our results provide empirical support for the theoretical prediction that cooperation can be maintained in non-kin-based dynamical societies via social assortment by cooperativeness.

Given our finding of assortment by cooperativeness in a real-world population, we might ask how this assortment is achieved. The assortment observed in the HP population could potentially arise passively as a by-product of assortment by other phenotypic traits, but our statistical tests did not support this for key traits that are known to influence social associations in guppies (sex [22], and body length [27,34]). Alternatively, there may be active mechanisms at play, where an individual's preference for or avoidance of others depends directly on their cooperativeness, and evidence implies that guppies use such strategies [23]. This does not necessarily require extensive cognitive abilities—theory shows that assortment of individuals can result from simple strategies of social partner choice. An example is the *walk-away* strategy, where an individual that experiences defection leaves the defector(s) and seeks out a new social environment, and computer modelling has shown that when this strategy is adopted, cooperation can be maintained via assortment of cooperators [39,40]. The *walk-away* strategy only requires assessing the cooperativeness of current inspection partners and subsequently reacting to this. Individuals adopting this strategy are therefore not limited by cognitively demanding book-keeping of the cooperativeness levels of others. Walk-away dynamics could thus potentially lie behind the assortment observed in the HP population, but further work is needed to draw conclusions regarding how assortment by cooperativeness comes about in guppies.

Our significant results of assortment for the HP population imply that individuals with greater similarity in cooperativeness have stronger ties to each other. We might expect this to be driven by strong ties between individuals with high cooperativeness, as they may gain more from building strong ties to each other than less cooperative individuals. However, we did not find any evidence for this, as there was no significant correlation between the summed cooperativeness of dyads and their edge weight. Intriguingly, it thus seems that the observed assortment by cooperativeness is caused by individuals of both higher and lower cooperativeness having strong ties to individuals of cooperativeness similar to their own. This result is similar to that of a study in humans, which found that individuals of both higher and lower cooperativeness were more likely to be tied to others with a similar cooperative propensity in a hunter–gatherer population [46]. Future studies can elucidate drivers of this pattern.

While both sexes in the HP population were assorted by cooperativeness, the results suggest a difference between sexes in the nature of the assortment: assortment in females came about by individuals of similar cooperativeness being more likely to be connected and having stronger ties to each other than expected, while in males only the presence, but not the strength of ties, contributed significantly to assortment. While the lower sample size for males could play a role for this difference, it may also be related to general differences between the sexes in social behaviour, with males moving between shoals more often than females to maximize the number of mating partners they encounter [27]. Interestingly, the Aripo HP population was the only one of the four HP populations tested that showed significant repeatability of cooperativeness for males, and the finding of male assortment by cooperativeness in the HP population may therefore be an exception rather than a rule. Without repeatability, males in the other populations cannot be said to have a cooperative phenotype by which to assort; however, given that repeatability in males in multiple cases approached significance, it is possible that repeatability is generally present in males, but that it is lower and more difficult to detect than in females. In any case, our results suggest that assortment by cooperativeness in guppies is of less importance in males than in females.

The lack of large predatory fish in low-predation guppy populations means that predator inspection has less importance for survival in these populations [13]. This led us to predict little or no assortment by cooperativeness in the LP population. Although assortment by cooperativeness in low-predation populations might still lead to slight fitness benefits, it is likely to be diluted by assortment by other traits [22,27,34,47–49]. Individuals are likely to face trade-offs when deciding whom to associate with and whom to avoid, and the final decision should depend on the relative importance of the various social partner traits for the fitness of the individual making the decision. The impact of the cooperativeness of social partners on individual social decisions is therefore likely to increase with higher predation pressure, and assortment by cooperativeness should therefore be primarily found in high-predation populations. In the LP population, we found that the social network was assorted by both body length and sex. These traits were related to cooperative tendency in this population, and assortment by these traits may therefore not be so disruptive for assortment by cooperativeness, but other traits not measured here are also known to influence association decisions and social assortment in guppies (e.g. familiarity [47] and attractiveness [48,49]). In the LP population, assortment by traits other than cooperativeness may be more important for fitness and may therefore have been prioritized at the expense of assorting by cooperativeness.

Most studies of animal social networks focus on a single population [12,50]. Here, we report the results quantifying the social network structure of two populations that differ in their ecological conditions. Although our findings provide the first insight into how assortment by cooperativeness may differ among predation regimes in guppies, a limitation here is that we do not have replication of populations within regimes. Our main result is the finding of assortment by cooperativeness in a wild animal population, and we consider the lack of assortment by cooperativeness in the LP population an intriguing finding, which illustrates the possibility of using intraspecific population comparisons to investigate mechanisms for the maintenance of cooperation. Further work is needed to determine the correlation between social assortment by cooperativeness and the ecological environment, and we look forward to future work in this area.

While the current study provides evidence that assortment by cooperative phenotypes can support cooperation in guppies, we know from previous work that guppies respond conditionally to the behaviour of others in cooperative contexts, by adjusting their cooperativeness to that of inspection partners to some extent. This includes both current and previous partners [15,17]. For example, a recent study found that guppies were more cooperative when their current inspection partner was more cooperative, and found population-dependent effects of previous inspection partners on levels of cooperativeness exhibited in subsequent inspections [17]. Thus, assortment by cooperativeness and conditional strategies of cooperation are likely to be concurrently contributing to the maintenance of cooperation in guppies, and they may interact if adjustment to partners affects longer-term individual levels of cooperativeness, potentially leading to a conformity effect [51]. This highlights that different theoretical explanations for non-kin cooperation are not mutually exclusive and their proposed mechanisms can operate simultaneously. To comprehensively understand the maintenance of non-kin cooperation in the real world therefore requires not only the determination of which maintenance mechanisms apply in real-world populations, but also their relative contributions.

Social assortment by cooperativeness constitutes a route to non-kin cooperation that does not necessarily require extensive cognitive capacities and therefore could be important in many species, but it has nevertheless been largely overlooked in empirical research. Our study provides empirical evidence that wild guppies show cooperative phenotypes and that they assort by these phenotypes when the expected impact of cooperative contexts on fitness is high. This supports the proposal that assortment by cooperativeness could play an important role for the evolution and persistence of cooperation among non-kin in nature. More empirical studies on assortment by cooperativeness in natural populations are eagerly anticipated.

## Supplementary Material

Appendix A

## Supplementary Material

Appendix B

## Supplementary Material

Appendix C

## Supplementary Material

Appendix D

## Data accessibility

The data used in this study are available at the Dryad Digital Repository: http://dx.doi.org/10.5061/dryad.js446q8 [52].
