# Supplementary material for: Evolution of non-kin cooperation: social assortment by cooperative phenotype in guppies
Source: R Soc Open Sci. 2018 Dec 26;6(1):181493. doi: 10.1098/rsos.181493 (PMC6366236; doi:10.1098/rsos.181493)
Supplement: Appendix A [file rsos181493supp1.pdf]

APPENDIX A
Schematic drawing of test tank

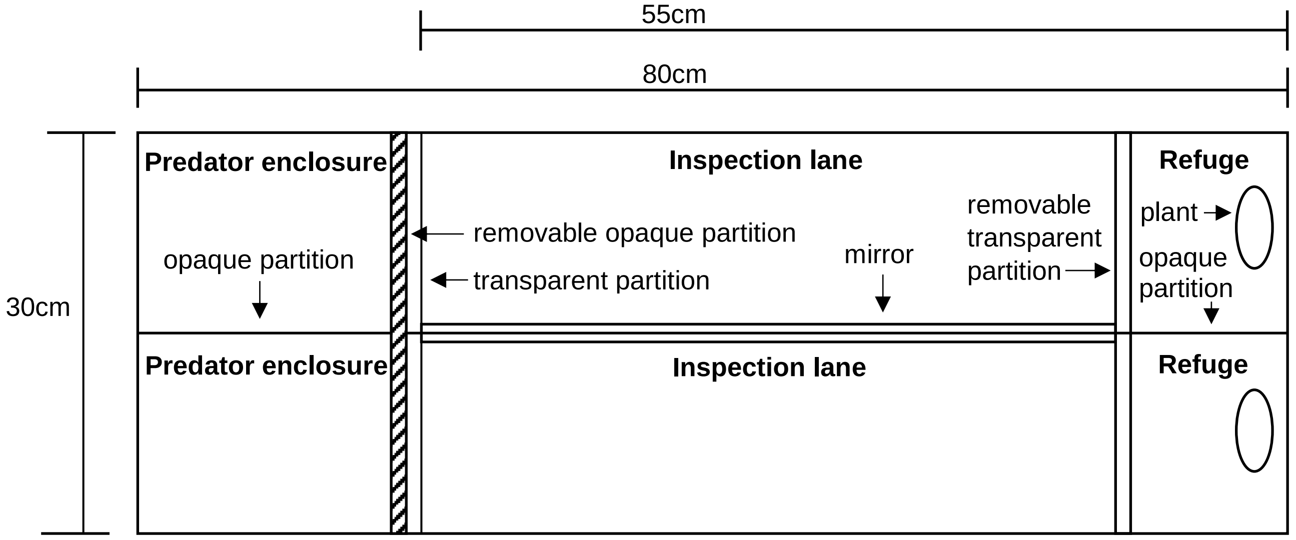

Schematic drawing of a predator inspection test tank. The tank is divided lengthwise into two identical, fully separated test arenas.
