# Supplementary material for: Evolution of non-kin cooperation: social assortment by cooperative phenotype in guppies
Source: R Soc Open Sci. 2018 Dec 26;6(1):181493. doi: 10.1098/rsos.181493 (PMC6366236; doi:10.1098/rsos.181493)
Supplement: Appendix B [file rsos181493supp2.pdf]

APPENDIX B

Validation of mirror trials as a means to measure cooperativeness

Methods

In order to validate the mirror cooperativeness scores (see main text), we quantified the relationship between cooperativeness scores from predator inspection trials with a mirror and with a live inspection partner, obtained from the same set of focal individuals. The setup and protocol of the trials with live partners was as in the mirror trials (described in the main text), except that there was no mirror, and the focal fish instead inspected with a live partner occupying the other lane in the test tank, which was visible through a transparent barrier. For each focal individual, four live-partner trials were run in total, each separated in time by one day. Each focal individual interacted with two partner individuals; the focal interacted with the first partner in trials 1 and 3, and the second partner in trials 2 and 4. Each partner fish was used with three focal fish. The use of each partner fish multiple times meant that effects of the partners on the behaviour of the focal individuals could be taken into account in the statistical analysis (as described below). Some focal individuals had data missing from some of the four trials and we limited the analysis to data where the focal had met the partner two times; that is, in the statistical tests described below, each focal individual contributed data from either trials 1&3, trials 2&4, or all four trials where possible. The partner fish were housed separately from the focal fish. Further details about the live-partner experiments can be found in Edenbrow *et al.* (2017).

For the live-partner trials, we used three measures of cooperativeness: (1) the focal fish's average distance from the refuge (corresponding to the measure used for the mirror trials); (2) the percentage of time the focal fish spent in the lead position ahead of the partner, i.e. closest to the predator; and (3) the average distance between the focal fish and its partner. In order to get general measures of cooperativeness for each focal individual across its live-partner trials, we extracted least square means (lsmeans) for the focal individuals for each of the three cooperativeness measures with linear mixed models, using the R-packages *lme4* (Bates *et al.* 2015) and *lsmeans* (Lenth 2016). These models had the respective cooperativeness measure as the dependent variable, focal ID as a fixed factor, and partner ID and test arena as random factors. Using general linear models, we then investigated the relationship between these lsmeans and the overall cooperativeness scores from the mirror trials (i.e. the means of the *average distance from refuge* scores of the two mirror trials). We constructed a model for each of the three live-partner trial measures for the high predation and low predation regime respectively, with the lsmeans as dependent variables. To investigate potential differences between populations and sexes in the relationship between mirror cooperativeness and live-partner cooperativeness, we initially included interactions between mirror cooperativeness and population, and between mirror cooperativeness and sex. These two factors were subsequently removed if the interactions were not significant. For all models, the lsmeans were transformed by arcsine square root transformation to meet test assumptions.

Results

Interactions between cooperativeness from the mirror trials and the two factors *sex* and *population* were not significant for any of the models, and these factors were therefore removed to allow for assessment of general relationships between mirror cooperativeness and live-partner cooperativeness. Cooperativeness quantified using a mirror was a significant predictor of cooperativeness shown with live-partners in both predation regimes (see table and figure below). Mirror cooperativeness was significantly positively correlated with the *average distance from refuge* lsmeans measure and the *percentage of time in lead position* lsmeans measure for both predation regimes. For the low predation regime, mirror cooperativeness was negatively correlated with the *average distance between the pair* lsmeans measure, while there was no significant correlation with this measure in the high predation regime.

Results of tests for relationships between cooperativeness measured in predator inspection trials where the focal fish inspected with their own mirror image, and cooperativeness from trials where the focal fish inspected with a live inspection partner. For the three live-partner cooperativeness measures we used least square means (lsmeans) derived from models that accounted for partner identity (see *Methods* above). Significant p-values are in bold.

Predation regime	Live-partner cooperativeness measure (lsmeans)	n	R^2		Estimate	Std. error	p
high predation	average distance from refuge	344	0.13	intercept	0.511793	0.021895	< **0.0001**
				mirror cooperativeness	0.000694	0.000096	< **0.0001**
	percent time in lead position	344	0.12	intercept	0.482086	0.028115	< **0.0001**
				mirror cooperativeness	0.000836	0.000124	< **0.0001**
	average distance between pair	344	0.0014	intercept	0.437700	0.014500	< **0.0001**
				mirror cooperativeness	0.000044	0.000064	0.4920
low predation	average distance from refuge	353	0.14	intercept	0.577500	0.027830	< **0.0001**
				mirror cooperativeness	0.000713	0.000095	< **0.0001**
	percent time in lead position	353	0.071	intercept	0.553300	0.026110	< **0.0001**
				mirror cooperativeness	0.000462	0.000089	< **0.0001**
	average distance between pair	353	0.088	intercept	0.540600	0.015450	< **0.0001**
				mirror cooperativeness	-0.000309	0.000053	< **0.0001**

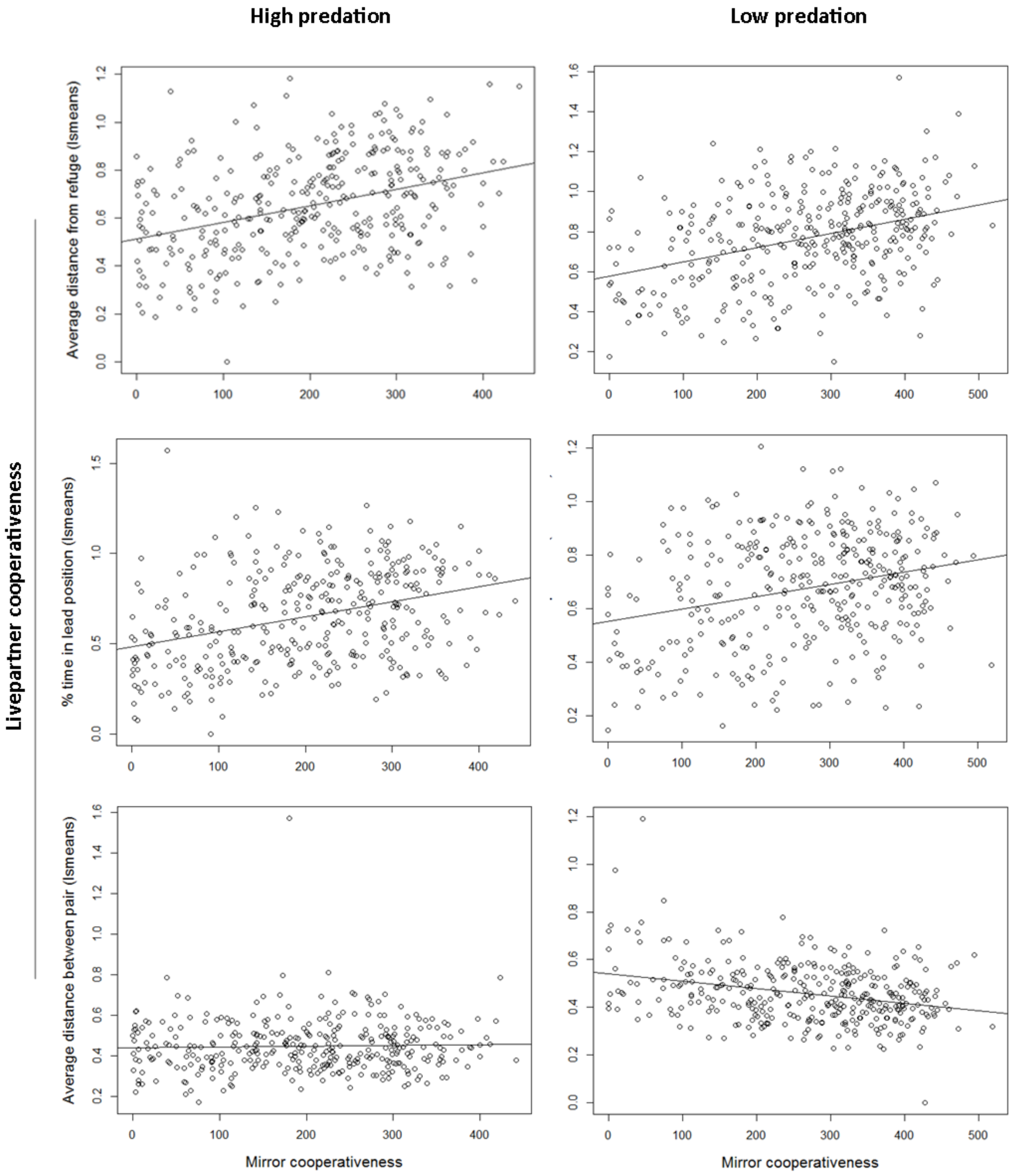

Relationships between cooperativeness measured in predator inspection trials where the focal fish inspected with its mirror image (mirror cooperativeness), and three measures of cooperativeness from trials where the focal fish inspected with a live partner (live-partner cooperativeness), for the high and low predation regime. Live-partner cooperativeness data are transformed least square means (lsmeans) extracted from models that account for partner identity (see *Methods* above).

References

Bates D, Maechler M, Bolker B, Walker S. 2015 Fitting Linear Mixed-Effects Models Using lme4. *J. Stat. Softw.* **67**, 1-48.

Edenbrow M, Bleakley BH, Darden SK, Tyler CR, Ramnarine IW, Croft DP. 2017 The evolution of cooperation: interacting phenotypes among social partners. *Am. Nat.* **189**, 630-643. (doi:10.1086/691386)

Lenth RV. 2016 Least-Squares Means: The R Package lsmeans. *J. Stat. Softw.* **69**, 1-33.
