# Supplementary material for: Evolution of non-kin cooperation: social assortment by cooperative phenotype in guppies
Source: R Soc Open Sci. 2018 Dec 26;6(1):181493. doi: 10.1098/rsos.181493 (PMC6366236; doi:10.1098/rsos.181493)
Supplement: Appendix C [file rsos181493supp3.pdf]

APPENDIX C
Details of genetic analysis

Genotyping of two populations of Trinidadian guppies. HP = high predation population, LP = low predation population. See below for a list of the given references.

		HP				LP				
**Locus**	**Multiple x**	**Size range**	**Observed hetero-zygosity (HO)**	**Expected hetero-zygosity (HE)**	**Test for Hardy–Weinberg Equilibrium (HWE) p-value**	**Size range**	**Observed hetero-zygosity (HO)**	**Expected hetero-zygosity (HE)**	**Test for Hardy–Weinberg Equilibrium (HWE) p-value**	**References**
G43	1	232-290	0.826	0.971	0.0446	282-392	0.625	0.860	<0.0001	Shen et al. (2007)
G289	1	280-316	0.767	0.825	0.4568	258-304	0.623	0.618	0.6388	Shen et al. (2007)
Pr171	1	163-369	0.395	0.693	<0.0001	253-365	0.703	0.811	<0.0001	Becher et al. (2002)
Pr39	1	148-190	0.701	0.790	0.0625	160-182	0.419	0.422	0.8021	Becher et al. (2002)
Pr80	1	133-215	0.678	0.777	0.0161	141-235	0.473	0.495	0.5386	Becher et al. (2002)
Pr92	1	157-175	0.628	0.564	0.3766	155-169	0.235	0.228	0.1146	Becher et al. (2002)
G183	2	231-293	0.679	0.704	0.1003	235-265	0.376	0.363	0.9762	Shen et al. (2007)
Pre8	2	182-346	0.718	0.894	<0.0001	180-336	0.714	0.720	0.4242	Paterson et al. (2005)
Pre15	2	183-335	0.918	0.947	0.4195	192-181	0.838	0.868	0.7069	Paterson et al. (2005)
Pre26	2	102-228	0.810	0.932	0.0113	110-216	0.765	0.758	0.3992	Paterson et al. (2005)
Pret69	2	110-174	0.506	0.583	0.614	110-178	0.507	0.461	0.8565	Watanabe et al. (2003)
AGAT11	3	212-345	0.906	0.952	0.0526	149-335	0.932	0.928	0.1958	Olendorf et al. (2004)
PP_5	3	161-305	0.953	0.955	0.1533	167-265	0.851	0.875	0.0453	Nater et al. (2008)
PP_H2	3	226-452	0.783	0.948	<0.0001	360-462	0.791	0.943	0.0065	Nater et al. (2008)
Pre17	3	139-205	0.423	0.846	<0.0001	93-243	0.427	0.597	<0.0001	Paterson et al. (2005)
Pre9	3	135-255	0.826	0.902	0.0044	163-235	0.628	0.657	0.2574	Paterson et al. (2005)

References

Becher SA, Russell ST, Magurran AE. 2002 Isolation and characterization of polymorphic microsatellites in the Trinidadian guppy (*Poecilia reticulata*). *Mol. Ecol. Notes* **2**, 456–458.

Nater A, Krützen M, Lindholm AK. 2008 Development of polymorphic microsatellite markers for the livebearing fish *Poecilia parae*. *Mol. Ecol. Resour.* **8**, 857–860.

Olendorf R, Reudi B, Hughes KA. 2004 Primers for 12 polymorphic microsatellite DNA loci from the guppy (*Poecilia reticulata*). *Mol. Ecol. Notes* **4**, 668–671.

Paterson IG, Crispo E, Kinnison MT, Hendry AP, Bentzen P. 2005 Characterization of tetranucleotide microsatellite markers in the guppy (*Poecilia reticulata*). *Mol. Ecol. Notes* **5**, 269–271.

Shen X, Yang G, Liao M. 2007 Development of 51 genomic microsatellite DNA markers of guppy (*Poecilia reticulata*) and their application in closely related species. *Mol. Ecol. Notes* **7**, 302–306.

Watanabe T, Yoshida M, Nakajima M, Taniguchi N. 2003 Isolation and characterization of 43 microsatellite DNA markers for guppy (*Poecilia reticulata*). *Mol. Ecol. Notes* **3**, 487–490.
