# Supplementary material for: Evolution of non-kin cooperation: social assortment by cooperative phenotype in guppies
Source: R Soc Open Sci. 2018 Dec 26;6(1):181493. doi: 10.1098/rsos.181493 (PMC6366236; doi:10.1098/rsos.181493)
Supplement: Appendix D [file rsos181493supp4.pdf]

APPENDIX D
Results of tests for social assortment by cooperativeness in tabular form

Weighted assortment. Results of tests for assortment by cooperativeness based on edge weights (r^w), for two wild guppy populations living under high (HP) and low (LP) predation pressure. Significant p-values are in bold.

Population	Individuals included	n	r^w from real network	Median r^w from permuted networks	p
HP	all	61	0.09	-0.03	**0.0004**
	females	40	0.12	-0.03	**0.0010**
	males	21	0.02	-0.10	0.0888
LP	all	102	0.00	0.00	0.7800
	females	55	-0.08	-0.03	0.3310
	males	47	-0.07	-0.03	0.5916

Unweighted assortment. Results of tests for assortment by cooperativeness based on the presence and absence of edges (r^u), for two wild guppy populations living under high (HP) and low (LP) predation pressure. Significant p-values are in bold.

Population	Individuals included	n	r^u from real network	Median r^u from permuted networks	p
HP	all	61	0.08	-0.02	**0.0004**
	females	40	0.09	-0.03	**0.0024**
	males	21	0.05	-0.08	**0.0412**
LP	all	102	0.02	-0.01	0.3500
	females	55	-0.06	-0.03	0.5286
	males	47	-0.06	-0.02	0.5982
